# Persistence Criteria for Susceptibility Genes for Schizophrenia: a Discussion from an Evolutionary Viewpoint

**DOI:** 10.1371/journal.pone.0007799

**Published:** 2009-11-11

**Authors:** Nagafumi Doi, Yoko Hoshi, Masanari Itokawa, Chie Usui, Takeo Yoshikawa, Hirokazu Tachikawa

**Affiliations:** 1 Department of Psychiatry, Ibaraki Prefectural Tomobe Hospital, Kasama-shi, Ibaraki, Japan; 2 Integrated Neuroscience Research Team, Tokyo Institute of Psychiatry, Kamikitazawa, Setagaya-ku, Tokyo, Japan; 3 Schizophrenia Research Project, Tokyo Institute of Psychiatry, Kamikitazawa, Setagaya-ku, Tokyo, Japan; 4 Department of Psychiatry, Faculty of Medicine, Juntendo University, Tokyo, Japan; 5 Laboratory for Molecular Psychiatry, RIKEN Brain Science Institute, Hirosawa, Wako, Saitama, Japan; 6 Department of Psychiatry, Graduate School of Comprehensive Human Science, Tsukuba University, Tsukuba, Ibaraki, Japan; Ohio State University Medical Center, United States of America

## Abstract

**Background:**

The central paradox of schizophrenia genetics is that susceptibility genes are preserved in the human gene-pool against a strong negative selection pressure. Substantial evidence of epidemiology suggests that nuclear susceptibility genes, if present, should be sustained by mutation-selection balance without heterozygote advantage. Therefore, putative nuclear susceptibility genes for schizophrenia should meet special conditions for the persistence of the disease as well as the condition of bearing a positive association with the disease.

**Methodology/Principal Findings:**

We deduced two criteria that every nuclear susceptibility gene for schizophrenia should fulfill for the persistence of the disease under general assumptions of the multifactorial threshold model. The first criterion demands an upper limit of the case-control difference of the allele frequencies, which is determined by the mutation rate at the locus, and the prevalence and the selection coefficient of the disease. The second criterion demands an upper limit of odds ratio for a given allele frequency in the unaffected population. When we examined the top 30 genes at SZGene and the recently reported common variants on chromosome 6p with the criteria using the epidemiological data in a large-sampled Finnish cohort study, it was suggested that most of these are unlikely to confer susceptibility to schizophrenia. The criteria predict that the common disease/common variant hypothesis is unlikely to fit schizophrenia and that nuclear susceptibility genes of moderate effects for schizophrenia, if present, are limited to ‘rare variants’, ‘very common variants’, or variants with exceptionally high mutation rates.

**Conclusions/Significance:**

If we assume the nuclear DNA model for schizophrenia, it should have many susceptibility genes of exceptionally high mutation rates; alternatively, it should have many disease-associated resistance genes of standard mutation rates on different chromosomes. On the other hand, the epidemiological data show that pathogenic genes, if located in the mitochondrial DNA, could persist through sex-related mechanisms.

## Introduction

While the development of genomics technology, coupled with sophisticated designs of linkage and association studies, is opening up new opportunities of genetics research of complex diseases, it may still be important to view the study of human disease from an epidemiological perspective [1]. The aim of this paper is to view the recent findings of molecular genetics of schizophrenia (SZ) and to examine the peculiarity of the genetic basis of SZ from an epidemiological standpoint.

SZ is a common deleterious psychosis with high heritability (80–85%), which manifests typically in adolescence or early adulthood [2]. SZ crosses all cultures at a relatively high prevalence (0.5–1%) [2], [3], and seems to be an ancient condition. The incidence of SZ, at the macro-level, varies within narrow limits [3], and appears to be stable across generations in several countries [4], [5]. On the other hand, it has been well documented that patients with SZ have a remarkably reduced reproductive fitness (0.3–0.8 as compared with the value in the normal population; the reduction is more pronounced in male patients) [6]–[17]. Then how can a pathogenic gene predisposing to SZ persist against a strong negative selection pressure? This ‘persistence problem’ has puzzled scientists for long years [18]–[20].

From an evolutionary viewpoint, four explanations are possible [18], [20]: (i) mutation-selection balance, (ii) heterozygote advantage (balancing selection), (iii) negative frequency-dependent selection, and (iv) ancestral neutrality.

‘Ancestral neutrality’ assumes that reproductive fitness of affected individuals and/or their relatives was higher in ancient environments and that selection coefficients of pathogenic alleles were close to zero. Because the effective population size in ancient times might be much smaller than now, pathogenic but neutral alleles could have been fixed by genetic drift. While this hypothesis explains that SZ has not been extinct in the long human history, ancestral neutrality itself provides no explanation for the apparently stable incidence of the disease across generations today; although ‘ancestral neutrality’ might be plausible, it needs another mechanism to account for the persistence of the disease in modern environments, where the effective population size has been expanded and the influence of negative selection pressure may be much stronger than ever before.

‘Negative frequency-dependent selection’ explains the persistence only when the fitness of the affected individuals increases as the prevalence in the general population decreases, which seems not to be the case with SZ.

‘Heterozygote advantage’ assumes that the susceptibility alleles increase the fitness of the unaffected gene carriers, thereby sustaining the gene frequencies. This line of explanations include: (i) physiological advantage (resistance to shock, infections, and poor nutrition etc.) [21], (ii) creative intelligence [22] or a higher trait creativity including ‘everyday creativity’ [23], and (iii) a higher sexual activity and/or attractiveness [24]. Since the unaffected siblings of the patients are expected to share pathogenic genes, those hypotheses need two lines of confirmation: (a) that the unaffected siblings of the patients have such advantages, and (b) that such advantages really contribute to sufficiently increase their reproductive fitness.

Some of those hypotheses seem to gain the confirmation (a). For example, Kinney et al. [23], in a well designed and methodologically sophisticated study, showed that an advantage of everyday creativity was linked to a subtle clinical picture (schizotypal signs) in a non-psychotic sample of SZ offspring.

However, those hypotheses lack the confirmation (b) in the nuclear DNA (ncDNA) model; those hypotheses, although theoretically plausible and fascinating, have not been supported by most epidemiological studies, which show a decreased reproductive fitness of the unaffected siblings of the patients [14], [16], [17], [25]–[28]. Haukka et al. [17], in a large-sampled cohort study, showed an increased reproductive fitness of unaffected *female* siblings of patients with SZ. However, this statistically higher fertility of the female siblings (1.033) was not large enough to compensate for the gene loss due to the decreased reproductive fitness of the patients (0.346) and their male siblings (0.950) in the ncDNA model. More recently, Svensson et al. [29], in a large-sampled three generation cohort study, did not find an increased fertility among parents, siblings or offspring of patients with SZ (except for a slightly and not significantly increased fertility ∼1.02 in healthy female siblings).

Thus, if we assume the ncDNA model for SZ, the remaining possibility is the mechanism of mutation-selection balance without heterozygote advantage. (Keller and Miller [20] comprehensively discussed this problem, leading to a similar conclusion. The difference from our argument is that Keller and Miller overlooked the possibility of ‘ancestral heterozygote advantage’ and discussed against ancestral neutrality.) Therefore, loss of the risk alleles due to the decreased reproductive fitness of the patients should be balanced by *de novo* mutation in each risk locus. A nuclear gene for SZ should meet this ‘persistence condition’ in addition to the condition of bearing a significant association with SZ. This simple and essential principle has been overlooked in SZ genetics.

Here we deduce two criteria that a nuclear susceptibility gene for SZ should fulfill for the persistence of the disease under general assumptions of multifactorial threshold model, and present their implications for genetic association studies and genetic models for SZ using the epidemiological data in a large-sampled Finish cohort study.

## Results

We deduced a series of criteria (‘persistence criteria’) that every nuclear susceptibility gene for SZ should fulfill for the persistence of the disease against a strong negative selection pressure. While the association condition between a risk allele and the disease demands the lower limit zero of the case-control difference of the allele frequencies (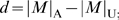 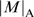 = allele frequency in the affected population, 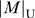 = allele frequency in the unaffected population), the first criterion demands an upper limit 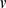 of the difference, which is determined by the prevalence of the disease (*p*), the selection coefficient of the disease (*s*), and the mutation rate at the locus (*μ*). Thus we have: 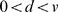, where 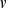 is defined by 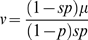. The second criterion derived from the first gives an upper limit of odds ratio (*OR*) of the pathogenic allele for a given allele frequency in the unaffected population. Since the association condition demands 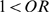, we have: 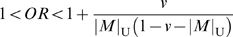 for 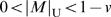.

Since mutation rates of the putative risk loci are unknown, three versions of the persistence criteria are shown in the **Table 1**. The stronger version corresponds to the lowest mutation rate 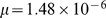 per locus per generation (for mutation rates see **section 4 in** **Method**) while the weaker version corresponds to the highest 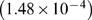 and the standard version corresponds to the average 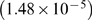.

**Table 1:** Three versions of persistence criteria.

	Stronger version	Standard version	Weaker version
*μ*	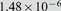	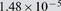	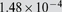
*ν*	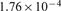	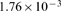	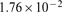
Criterion A	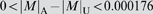	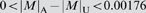	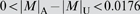
Criterion B	For 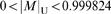 , 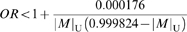	For 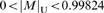 , 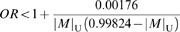	For 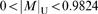 , 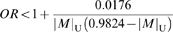

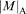
: Allele frequency in the affected population,

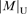
: Allele frequency in the unaffected population.

Because the estimated value of 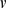 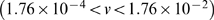 is remarkably small, the persistence criteria is very demanding. Among the 36 single nucleotide polymorphisms (SNPs) at SZGene [30] that have significant *P* values (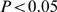) in the meta-analyses, only 9 SNPs fulfill the weaker version of the criterion A (**Table 2**): the G-allele of rs1801028 (***DRD2***), the C-allele of rs1327175 (***PLXNA2***), the A-allele of rs9922369 (***RPGRIP1L***), the A-allele of rs2391191 (***DAOA***), the C-allele of rs35753505 (***NRG1***), the G-allele of rs4680 (***COMT***), the T-allele of rs737865 (***COMT***), the T-allele of rs1011313 (***DTNBP1***), and the A allele of rs3213207 (***DTNBP1***). None of these SNPs meet the standard version of the criteria. Therefore, these SNPs cannot meet the persistence criteria unless they have the highest mutation rate.

**Table 2:** Top polymorphisms in Top 30 genes at SZGene [30] (August 10, 2009).

Genes and SNPs	Allele (minor/major)	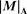 (sample size)	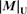 (sample size)	*P*-value	*OR*	*d*
*1. DISC1*						
rs3737597	A*/G	0.07881 (N = 1,142)	0.05231 (N = 1,797)	**0.000245**	1.4	0.0265
*2. SLC18A1*						
rs2270641	C*/A	0.31818 (N = 759)	0.28022 (N = 885)	0.0614	1.63	0.0380
*3. GABRB2*: none						
*4. * ***DRD2***						
rs1079597 (Taql-B)	A/G*	0.81325 (N = 830)	0.78273 (N = 803)	**0.0229**	1.37	0.0315
rs6277	C*/T	0.50412 (N = 3,159)	0.46080 (N = 4,043)	**0.00000199**	1.37	0.0433
**rs1801028**	G*/C	0.03337 (N = 6,173)	0.02643 (N = 7,908)	**0.00323**	1.22	**0.0069**
rs6275	T*/C	0.33862 (N = 2,903)	0.31100 (N = 3,336)	**0.00198**	1.15	0.0276
5. GWA 10q26.13						
rs17101921	A*/G	0.06667 (N = 7,447)	0.04318 (N = 13,039)	**0.00000000**	1.28	0.0235
*6. AKT1*						
rs3803300	A*/G	0.33705 (N = 2,645)	0.31460 (N = 2,999)	**0.0257**	1.05	0.0225
*7. GRIN2B*						
rs1019385	T/G*	0.56041 (N = 687)	0.48846 (N = 650)	**0.00050**	1.33	0.0720
rs7301328	G*/C	0.44256 (N = 1,088)	0.40845 (N = 994)	0.0862	1.17	0.0341
*8. DGCR2*						
rs2073776	A*/G	0.39824 (N = 2,727)	0.37117 (N = 3,004)	**0.010**	1.14	0.0271
*9. * ***PLXNA2***						
**rs1327175**	G/C*	0.92840 (N = 1,711)	0.91243 (N = 1,770)	**0.043**	1.32	**0.0160**
*10. * ***RPGRIP1L***						
**rs9922369**	A*/G	0.04221 (N = 5,474)	0.03437 (N = 10,823)	**0.0014**	1.3	**0.0078**
*11. TPH1*						
rs1800532	A*/C	0.50726 (N = 1,239)	0.45052 (N = 1,708)	**0.0000799**	1.25	0.0567
*12. DRD4*						
120-bp TR	S/L*	0.80421 (N = 1,236)	0.76397 (N = 1,199)	**0.00380**	1.23	0.0402
rs1800955	C*/T	0.41964 (N = 2,128)	0.39823 (N = 2,206)	0.0653	1.13	0.0231
*13. * ***DAOA***						
rs3916971	T/C*	0.56220 (N = 844)	0.52115 (N = 922)	**0.045**	1.19	0.0411
rs778294	T/C*	0.78375 (N = 6,444)	0.77250 (N = 7,677)	0.069	1.04	**0.0113**
**rs2391191 (M15)**	A*/G	0.50063 (N = 8,692)	0.48820 (N = 10,680)	**0.029**	1.01	**0.0124**
*14.* GWA 11p14.1						
rs1602565	C*/T	0.14240 (N = 7,170)	0.12112 (N = 12,611)	**0.00000001**	1.16	0.0213
*15. DRD1*: none						
*16. HTR2A*						
rs6311	A/*G	0.44847 (N = 2,678)	0.41784 (N = 2,964)	**0.00457**	1.16	0.0306
*17. RELN*						
rs7341475	A/G*	0.85477 (N = 3,009)	0.82569 (N = 7,045)	**0.00000283**	1.14	0.0291
*18. APOE*	e2/3/4*	0.12061 (N = 2,931)	0.10257 (N = 5,065)	**0.0135**	1.09	0.0181
*19. * ***NRG1***						
rs2439272	A/G*	0.64395 (N = 2,935)	0.61284 (N = 2,797)	**0.00101**	1.18	0.0312
**rs35753505**	C*/T	0.42656 (N = 9.082)	0.41024 (N = 9,921)	**0.00658**	1.04	**0.0163**
rs473376	G*/A	0.17252 (N = 3,701)	0.14611 (N = 4,589)	**0.0000435**	1.08	0.0264
*20. IL1B*						
rs1143634	T/C*	0.83626 (N = 1,197)	0.81951 (N = 1,435)	0.0564	1.06	**0.0167**
*21. MTHFR*						
rs1801133	T*/C	0.34340 (N = 4,055)	0.31491 (N = 5,535)	**0.000135**	1.14	0.0341
*22. * ***COMT***						
**rs4680**	A/G*	0.58316 (N = 13,282)	0.56823 (N = 17,580)	**0.000108**	1.02	**0.0149**
**rs737865**	C/T*	0.69100 (N = 6,288)	0.67468 (N = 9,131)	**0.00320**	0.95	**0.0163**
*23. HP*						
Hp1/2	1/2*	0.62296 (N = 1,346)	0.59291 (N = 2,018)	**0.0443**	1.14	0.0300
*24. DAO*						
rs2111902	G*/T	0.39094 (N = 2,517)	0.36807 (N = 2,960)	**0.0455**	1.07	0.0229
rs3741775	C/G*	0.57980 (N = 2,514)	0.55542 (N = 2,959)	**0.0218**	1.09	0.0244
rs3918346	A*/G	0.35145 (N = 2,521)	0.32957 (N = 2,966)	**0.0463**	1.05	0.0219
rs4623951	C/T*	0.78378 (N = 1,509)	0.67883 (N = 1,521)	0.0915	1.14	0.0249
*25. TP53*						
rs1042522	C*/G	0.39880 (N = 1,418)	0.36879 (N = 1,410)	0.0675	1.13	0.0300
*26. ZNF804A*						
rs1344706	G/T*	0.59933 (N = 7,183)	0.58402 (N = 12,663)	**0.0129**	1.12	0.0191
27. GWA 16p13.12						
rs71992086	T*/A	0.27009 (N = 7,179)	0.24558 (N = 12,623)	**0.00000039**	1.12	0.0245
*28. * ***DTNBP1***						
**rs1011313**	T*/C	0.11722 (N = 7,695)	0.10562 (N = 7,276)	**0.00652**	1.08	**0.0116**
rs1018381	T/*C	0.09666 (N = 4,940)	0.08727 (N = 4,927)	0.0763	1.11	**0.0094**
rs2619538(SNPA)	T*/A	0.49804 (N = 5,598)	0.47671 (N = 5,862)	**0.00758**	1	0.0213
**rs3213207(P1635)**	G/A*	0.90835 (N = 8,472)	0.89811 (N = 8,391)	**0.00694**	1.08	**0.0102**
*29. OPCML*						
rs3016384	T/C*	0.53882 (N = 7,187)	0.51744 (N = 12,675)	**0.000264**	1.08	0.0214
*30. RGS4*						
rs2661319 (SNP16)	A/G*	0.49313 (N = 8,010)	0.47446 (N = 9,183)	**0.00249**	1.08	0.0187

* alleles associated with SZ, 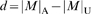.

SNPs with *P*-value less than 0.1 are listed.

None of the recently reported common SNPs on chromosome 6p22.1 associated with SZ [31] meet the weaker version of the criterion A (**Table 3**). Therefore, those common variants are unlikely to confer susceptibility to SZ unless they have exceptionally high mutation rates. The best imputed SNP in a recent genome-wide association study (GWAS) [32], which reached a genome-wide significance (the A-allele of rs3130297 on chromosome 6p; 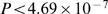), does not meet the weaker version of the criterion A (*d*>0.02; see Table 1 in the paper [32]). Therefore, this SNP is unlikely to contribute to risk of SZ unless it has an exceptionally high mutation rate. Similarly, none of the top 100 SNPs in a recent GWAS [32] fulfill the weaker version of the criterion A (see Table 1 in the paper [33]).

**Table 3:** Common variants on chromosome 6p22.1 associated with SZ [31].

rs ID	Allele (minor/major)	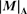	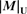	*P*-value	*OR*	*d*
rs6904071	A/G*	0.834	0.814	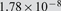	1.14–1.25	0.020
rs926300	T/A*	0.834	0.814	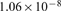	1.14–1.26	0.020
rs6913660	A/C*	0.836	0.816	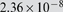	1.13–1.25	0.020
rs13219181	G/A*	0.837	0.817	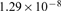	1.14–1.26	0.020
rs13194053	C/T*	0.838	0.818	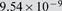	1.14–1.28	0.020
rs3800307	A/T*	0.817	0.795	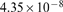	1.13–1.27	0.022
rs3800316	C/A*	0.771	0.743	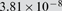	1.13–1.20	0.028

* alleles associated with schizophrenia, 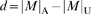.

Three of the 7 common SNPs associated with SZ in the latest GWAS [34] clearly do not meet the weaker version of the criterion B. The remaining 4 SNPs may fulfill the weaker version but not the standard version (see Table 1 in the paper [34]). Therefore, these 4 SNPs are unlikely to confer susceptibility to SZ unless they have the highest mutation rate.

*OR* for a given allele frequency in the unaffected population and the range of allele frequency in the unaffected population for a given *OR* calculated with the criterion B under three levels of mutation rate are presented in the **Tables 4** and **5**, respectively.

**Table 4:** *OR* vs. allele frequency in the unaffected population.

	Stronger version	Standard version	Weaker version
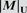	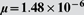	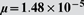	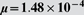
0.001	<1.18	<2.77	<17.9
0.01	<1.02	<1.18	<2.81
0.02	<1.009	<1.09	<1.92
0.05	<1.004	<1.04	<1.38
0.1	<1.002	<1.02	<1.20
0.3	<1.0009	<1.009	<1.09
0.5	<1.0008	<1.008	<1.08
0.7	<1.0009	<1.009	<1.09
0.9	<1.002	<1.02	<1.24
0.95	<1.004	<1.04	<1.58
0.98	<1.009	<1.10	<8.49
0.99	<1.02	<1.22	-*
0.999	<1.22	-*	-*

*The upper limit of *OR *is dependent on the allele frequency in the affected population:
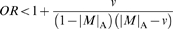

**Table 5:** Allele frequency in the unaffected population vs. *OR*.

	Stronger version	Standard version	Weaker version
*OR*	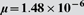	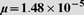	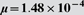
1.2	<0.0009, or >0.9989	<0.009, or >0.989	<0.10, or >0.883
1.5	<0.0004, or >0.9994	<0.004, or >0.994	<0.04, or >0.945
2.0	<0.0002, or >0.9995	<0.002, or >0.996	<0.02, or >0.964
3.0	<0.00009, or >0.9997	<0.0009, or >0.997	<0.009, or >0.973
5.0	<0.00005, or >0.9997	<0.0005, or >0.997	<0.005, or >0.978
10.0	<0.00002, or >0.9998	<0.0002, or >0.998	<0.002, or >0.980

Required sample sizes for association studies for a single allele and for GWAS are shown in the **Tables 6** and **7**, respectively. Powers of association study for a single allele and of GWAS with given sample sizes are shown in **Tables 8** and **9**. Surprisingly, the power of GWAS with a sample size as large as 100,000 case-control pairs to detect a common variant of a mutation rate not higher than the average is almost zero (**Table** **9**).

**Table 6:** Required sample size in an association study for a common variant.

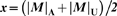	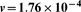	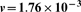	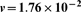
0.1 or 0.9			
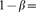 0.95	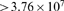	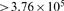	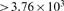
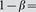 0.80	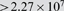	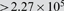	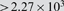
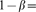 0.10	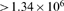	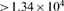	>134
0.2 or 0.8			
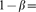 0.95	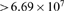	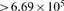	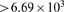
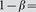 0.80	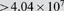	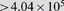	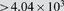
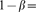 0.10	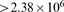	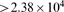	>238
0.3 or 0.7			
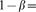 0.95	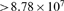	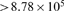	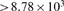
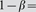 0.80	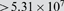	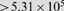	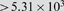
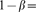 0.10	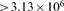	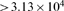	>313
0.4 or 0.6			
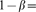 0.95	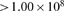	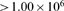	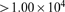
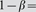 0.80	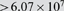	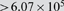	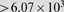
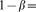 0.10	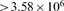	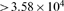	>358
0.5			
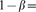 0.95	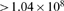	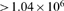	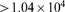
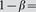 0.80	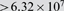	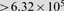	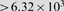
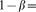 0.10	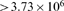	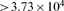	>373

Samples: *N* cases + *N* controls, 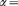
0.05, 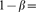
0.95, 0.8, 0.1.

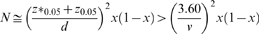
 for 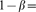
0.95.

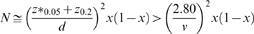
 for 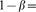
0.80.

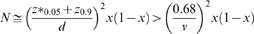
 for 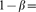
0.10.

**Table 7:** Required sample size in GWAS for SZ.

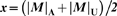	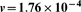	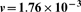	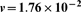
0.1 or 0.9			
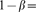 0.95	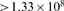	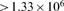	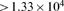
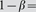 0.80	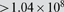	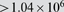	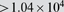
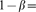 0.10	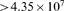	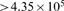	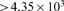
0.2 or 0.8			
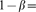 0.95	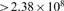	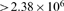	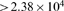
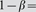 0.80	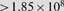	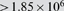	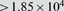
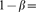 0.10	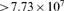	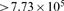	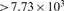
0.3 or 0.7			
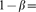 0.95	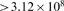	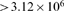	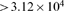
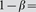 0.80	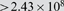	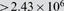	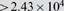
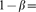 0.10	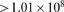	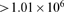	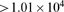
0.4 or 0.6			
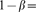 0.95	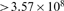	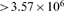	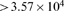
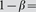 0.80	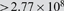	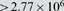	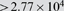
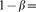 0.10	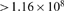	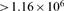	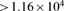
0.5			
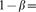 0.95	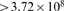	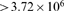	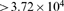
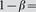 0.80	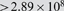	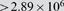	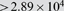
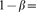 0.10	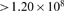	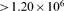	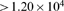

Samples: *N* cases + *N* controls, 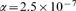, 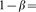
0.95, 0.8, 0.1.

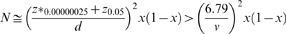
 for 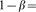
0.95.

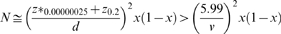
 for 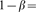
0.80.

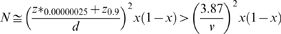
 for 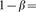
0.10.

**Table 8:** Power of association study for a single variant.

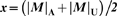	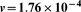	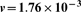	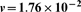
0.1 or 0.9			
*N* = 10,000	<0.03	<0.09	<1
*N* = 20,000	<0.04	<0.13	<1
*N* = 50,000	<0.04	<0.27	<1
0.2 or 0.8			
* N* = 10,000	<0.03	<0.07	<0.999
*N* = 20,000	<0.03	<0.10	<1
*N* = 50,000	<0.04	<0.17	<1
0.3 or 0.7			
*N* = 10,000	<0.03	<0.07	<0.99
*N* = 20,000	<0.03	<0.08	<0.9999
*N* = 50,000	<0.04	<0.14	<1
0.4 or 0.6			
*N* = 10,000	<0.03	<0.06	<0.95
*N* = 20,000	<0.03	<0.08	<0.9999
*N* = 50,000	<0.04	<0.13	<1
0.5			
*N* = 10,000	<0.03	<0.05	<0.95
*N* = 20,000	<0.03	<0.08	<0.999
*N* = 50,000	<0.04	<0.13	<1

Samples: *N* cases + *N* controls, 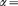
0.05.

Power: 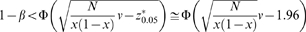.

**Table 9:** Power of GWAS for SZ.

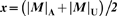	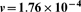	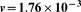	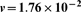
0.1 or 0.9			
* N* = 10,000	<0.000001	<0.00001	<0.76
*N* = 50,000	<0.000001	<0.0001	<1
*N* = 100,000	<0.000001	<0.001	<1
0.2 or 0.8			
* N* = 10,000	<0.000001	<0.00001	<0.23
*N* = 50,000	<0.000001	<0.0001	<1
*N* = 100,000	<0.000001	<0.001	<1
0.3 or 0.7			
* N* = 10,000	<0.000001	<0.00001	<0.10
*N* = 50,000	<0.000001	<0.0001	<0.9999
*N* = 100,000	<0.000001	<0.0001	<1
0.4 or 0.6			
* N* = 10,000	<0.000001	<0.00001	<0.07
*N* = 50,000	<0.000001	<0.0001	<0.999
*N* = 100,000	<0.000001	<0.0001	<1
0.5			
*N* = 10,000	<0.000001	<0.00001	<0.06
*N* = 50,000	<0.000001	<0.0001	<0.999
*N* = 100,000	<0.000001	<0.0001	<1

Samples: *N* cases + *N* controls, 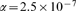.

Power: 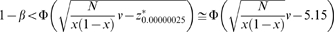.

## Discussion

**Figure 1:**
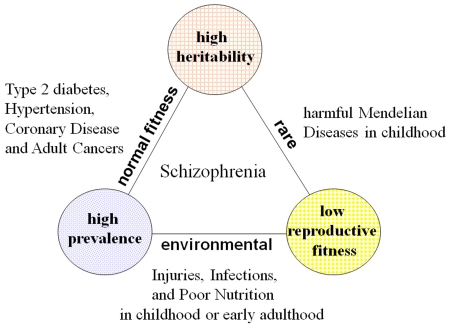
Devil's triangle of high heritability, high prevalence and low reproductive fitness. The three epidemiological properties-high heritability, high prevalence and low fitness- form a Devil's triangle; any combination of the two tends to exclude the third. In this triangle most diseases vanish except for schizophrenia.

The three epidemiological properties- high heritability, high prevalence and low reproductive fitness- form a Devil's triangle; any combination of the two tends to exclude the third, and in this triangle most diseases vanish except for SZ (**Figure 1**). Diseases with high heritability and high prevalence such as type 2 diabetes and adult cancers are late-onset diseases and may show almost normal reproductive fitness. Diseases with high prevalence and low reproductive fitness such as poor nutrition, severe injuries and infections in childhood or early adulthood are mainly due to the environmental factors. Diseases with low reproductive fitness and high heritability such as most harmful Mendelian diseases in childhood are rare. From this point of view, SZ, a disease with those three properties, may be unique and peculiar.

This peculiar epidemiological characteristic of the disease may put SZ in a unique position among the common diseases with genetic bases; it might be afforded, not surprisingly, by a unique and peculiar genetic basis. The persistence criteria, although with notable limitations such as assuming a large effective population size at equilibrium and random mating (see **Method**), may approximately describe the peculiarity of the genetic basis for SZ. Let us examine the peculiarity of SZ genetics with the persistence criteria.

### 1. The CD/CV hypothesis is unlikely to fit SZ

First, we can see that the common disease/common variant (CD/CV) hypothesis [35], [36] is unlikely to fit SZ. The standard version of the criterion B implies that the *OR* of every risk allele with a population frequency between 0.05 and 0.95 is less than 1.04 (**Table 4**). The weaker version implies that the *OR* of every risk allele with a population frequency between 0.04 and 0.945 is less than 1.50 (**Table 5**). Therefore, given the standard range of mutation rate (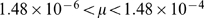), the effect size and the population frequency of a nuclear risk variant for SZ cannot simultaneously satisfy the expectations in the CD/CV hypothesis, in which common alleles at a handful of loci are assumed to interact to cause a common disease.

### 2. Nuclear risk variant for SZ of moderate effects, if present, should be either rare or very common

As previously mentioned, the persistence criteria argue against the CD/CV hypothesis. However, it does not necessarily mean that only the multiple rare variant model [37], [38] fits SZ. The standard version of the criterion B implies that the frequency of a pathogenic variant of a moderate effect (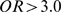) in the ncDNA, if present, should be either very low in the affected population (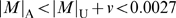) or very high in the normal population (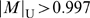) (**Table 5**). The weaker version implies that the frequency of a nuclear susceptibility variant of a moderate effect should be either low (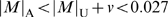) or high (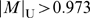) (**Table 5**). Thus we can see that given the standard range of mutation rate nuclear genes of moderate effects for SZ, if present, are limited to either ‘rare variants’ or ‘very common variants’.

‘Very common variants’ for a deleterious disease might seem at odds; how could variants associated with a deleterious disease ever have become so common in spite of the enormous cost the species should pay for?

Given a much smaller effective population size in ancient times, ‘ancestral heterozygote advantage’ and genetic drift, coupled with less pronounced reproductive disadvantage of the ancestral patients, could provide an explanation. Although the ancestral patients might also show a reduced reproductive fitness, the reproductive disadvantage could have been less pronounced in ancient environments because many patients could have children before the onset of their illness; individuals in ancient times might have their first children at a lower age (15–20 years = adolescence) than individuals in modern times (25–30 years; see **section 4 in** **Method**). Advantages of the unaffected siblings such as everyday creativity could better work to increase their reproductive fitness in ancient times than today. In addition, the effective population size might be much smaller in ancient times. Thus, susceptibility genes could have been neutral or almost neutral (selection coefficient 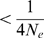; 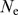 =  the effective population size) in ancient times. Then, pathogenic but neutral or almost neutral genes in ancient environments could be fixed at a high frequency close to 1 by genetic drift (because the effective population size might be much smaller and the effects of genetic drift might be predominant in ancient times) and can be sustained by mutation-selection balance today.

For the past two years several large-scaled association studies including GWAS for SZ have been reported [31]–[34], [39]–[45]. These reports have essentially ruled out the likelihood of a few common variants conferring the majority of SZ heritability. On the other hand, several groups have shown that both *de novo* and inherited rare variants including copy number variants (CNV) with high odds ratios are associated with SZ [46]–[51]. Although the roles of these rare variants in the pathogenesis of SZ remain unclear, these reports seem to be in line with the predictions of the persistence criteria. However, no reports have identified ‘very common variants’ associated with SZ to date.

### 3. The largest GWAS to date lacks the power to identify a common variant of the average mutation rate

The persistence criteria predict that common pathogenic variants, if present, can have only tiny effects. Nevertheless, identification of common pathogenic variants would be much more difficult than previously thought. The persistence criteria imply that *the sample size required in an association study for SZ with a given power depends on the mutation rate at the putative risk locus as well as the population frequency of the putative pathogenic variant*. Thus we can see that an enormous sample size is required to identify a common pathogenic variant of a standard mutation rate (**Table 6** and **7**). For example, more than the half of all the SZ patients in the world (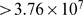; we assume here a total human population of 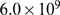 and a prevalence of 1%) and the same number of control subjects should be recruited to the association study to identify a common variant (population frequency: 0.1–0.9) at a putative risk locus of a mutation rate 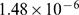 with a power 0.95 (**Table 6**). When the mutation rate is assumed to be average, more than one million case-control pairs are required to identify a common variant in a GWAS with a power 0.8 (**Table 7**).

Because the sample size of the largest GWAS and association studies to date is far less than 50,000 case-control pairs (**Tables 10** and **11**), those studies lack the power to identify a common pathogenic variant of the average mutation rate (**Tables 8** and **9**). The power of the GWAS to identify common variants of the highest mutation rate has merely reached to the level of ∼0.1 for the past two years (**Tables 7** and **9**).

**Table 10:** Pooled sample sizes in association studies for top 30 genes at SZGene [30].

Candidates	Cases (Caucasian)	Controls (Caucasian)	Cases (Total)	Controls (Total)
***1. DISC1***	5,762	7,449	8,006	9,697
***2. SLC18A1***	673	1,283	1,346	1,948
***3. GABRB2***	1,625	1,788	2,887	2,873
***4. DRD2***	8,291	11,436	10,915	14,259
**5. GWA 10q26.13**	5,666	11,174	7,531	13,039
***6. AKT1***	2,798	3,274	4,248	4,662
***7. GRIN2B***	737	704	1,765	1,680
***8. DGCR2***	1,195	1,384	5,549	5,771
***9. PLXNA2***	705	739	1,401	1,685
***10. RPGRIP1L***	5,526	10,969	5,526	10,969
***11. TPH1***	905	1,845	1,960	3,068
***12. DRD4***	4,027	5,684	7,070	8,307
***13. DAOA***	5,562	7,290	9,424	11,555
**14. GWA 11p14.1**	5,526	10,969	7,308	12,834
***15. DRD1***	1,303	1,917	1,502	2,213
***16. HTR2A***	8,226	8,809	10,907	11,284
***17. RELN***	3,705	8,301	4,711	9,340
***18. APOE***	2,624	4,646	4,693	7247
***19. NRG1***	7,069	9,494	12,995	15,091
***20. IL1B***	1,420	2,373	2,161	3,096
***21. MTHFR***	3,411	5,037	4,752	6,320
***22. COMT***	12,640	22,644	18,140	29,065
***23. HP***	1,300	1,966	1,863	2,492
***24. DAO***	1,953	2,427	3,120	3,585
***25. TP53***	383	443	1,418	1,410
***26. ZNF804A***	5,526	10,969	7,308	12,834
**27. GWA 16p13.12**	5,526	10,969	7,308	12,834
***28. DTNBP1***	8,306	9,902	10,392	11,756
***29. OPCML***	5,526	10,969	7,308	12,834
***30. RGS4***	7,756	8,983	10,466	11,711

**Table 11:** Sample sizes of GWAS for SZ to date.

Study	Population	# of SNPs	# of cases	# of controls
Mah, 2006	Caucasian, USA	25,494	320	325
Lenz, 2007	Caucasian, USA	439,511	178	144
Kirov, 2008	Caucasian, Bulgaria	433,680	574	1,753
Shifman, 2008	Caucasian, Israel	510, 552	660	2,771
O'Donovan, 2008	Mixed	362,532	7,308	12,834
Sullivan, 2008	Mixed, USA	492,900	738	733
Need, 2009	European origin	555,352	1,460	12,995
Stefasson, 2009	Europe	314,868	12,945	34,591
Shi, 2009	Mixed		8,008	19,077
The International Schizophrenia Consortium, 2009	Europe		3,322	3,587

### 4. Too strong association implies that the variants may not confer susceptibility

Since the criterion A demands a small upper limit of the case-control difference of the allele frequencies, too strong association imply that the allele may not confer susceptibility to SZ. Especially, common variants associated with SZ in an association study with a sample size smaller than the estimations in the **Tables 6** and **7** are unlikely to contribute to risk of SZ.

Let us consider the cases of the SNPs in the **Table 2**. Among the 36 SNPs that have significant *P* values in the meta-analyses at SZGene, 9 SNPs can fulfill the weaker version of the criteria only if they have the highest mutation rate. However, the remaining 27 SNPs cannot meet the criteria unless they have exceptionally high mutation rates (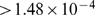). For example, the G-allele of rs1019385 (*GRIN2B*), which shows *P* = 0.0005 in the meta-analysis, cannot meet the criteria unless the mutation rate of the locus is higher than 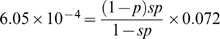. However, this value may be too high as compared with the upper limit of mutation rates on autosomes and X chromosome (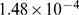). Alternatively, this SNP must be a protective or resistance gene (i.e. a gene elevating the carrier's fitness by reducing the liability to the disease as well as the severity of the disease).

It should be noted that high mutation rates (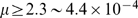) have been reported on human Y chromosome [52]. Therefore, common variants on Y chromosome or on the pseudoautosomal regions of X chromosome where abundant mutation could be supplied by synapsis and crossing over with Y chromosome, could meet the persistence criteria. In this case, however, putative risk loci would be highly polymorphic because of abundant mutation supply. Common CNVs also could meet the criteria, if they have extremely high mutation rates (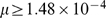).

In the future, with expansion of the sample size and pooled data, GWAS and meta-analyses may identify many more variants associated with SZ. While some of them may fulfill the persistence criteria, the others do not. Then, associated variants that do not fulfill the persistence criteria should be either susceptibility genes of exceptionally high mutation rates or resistance genes of standard mutation rates. Thus, in the near future, we are to choose one of the alternative cases: (1) a case in which SZ should have many susceptibility genes with tiny effects of exceptionally high mutation rates, or (2) a case in which SZ should have many resistance genes of standard mutation rates on different chromosomes associated with SZ itself. This may be the most peculiar aspect of SZ genetics that the persistence criteria predict.

### 4. Alternative direction for searching for SZ genes

We have discussed the peculiarity of SZ genetics under the assumption that the risk loci are located in the ncDNA. Now we shall remember that there is another possibility for the location of the risk loci.

Another possibility is that a pathogenic gene is located in the mitochondrial DNA (mtDNA), which shows a higher mutation rate than the ncDNA: 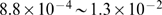 per locus per generation (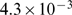 on average) [53].

Because mtDNAs are transmitted only through females, the mtDNA model could explain the persistence by a higher reproductive fitness of the unaffected *female* siblings of the patients (heterozygote advantage in this model) and/or a reduced male/female ratio in the offspring in the predisposed matrilineal pedigrees [54].

Interestingly, recent epidemiological studies have consistently shown that the reproductive fitness of the unaffected *female* siblings of the patients is slightly increased (1.02–1.08) [14], [16], [17], [29]. The epidemiological data by Haukka et al. [17] show that the slightly increased reproductive fitness of the unaffected female siblings of the patients (1.033), coupled with less pronounced reduced reproductive fitness of the *female* patients (0.46), is sufficient for the persistence of the disease in the mtDNA model.

Let us calculate 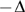, the cross-generational reduction of the frequency of females with the pathogenic mtDNA in the general population, using their epidemiological data (**Table 12**). At first we define several notations. 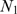: number of the normal female population in the first generation; 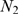: number of the female offspring of the normal female population; 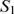: number of the unaffected female siblings of the patients in the first generation; 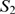: number of the female offspring of the unaffected female siblings of the patients; 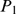: number of the female patients; 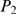: number of the female offspring of the female patients; *r* (0<*r*<1): proportion of the gene carriers in the normal female population in the first generation. Then number of the female gene carriers in the first generation is 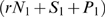 and 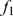, frequency of the female gene carriers in the first generation, is given by: 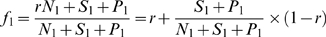. The expected number of the female gene carriers in the second generation is 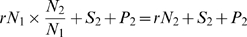 and 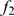, frequency of the female gene carriers in the second generation, is 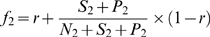. Therefore it follows: 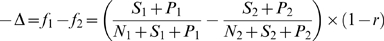



**Table 12:** Epidemiological data by Haukka et al. [17].

	*N*	*S*	*P*	*Total*	*(S+P)/Total*
# of females	410,093	11,873	4,784	426,750	0.03903
# of female children	366,460	10,969	1,917	379,346	0.03397
					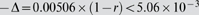

*N*: Normal females; *S*: Unaffected female siblings of patients; *P*: Female patients with SZ;

*r*: Proportion of the gene carriers in the normal population in the first generation (0<*r*<1);

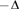
: Reduction of the frequency of females with the pathogenic mtDNA in the general population.

Thus we have: 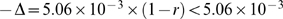 (**Table 12**). This implies that the gene loss can be balanced by *de novo* mutation in the mtDNA which occurs at a rate of 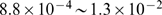 per locus per generation (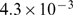 on average) [53]. Therefore the mildly elevated reproductive fitness of the unaffected female siblings of the patients is sufficient to sustain the gene frequency in the mtDNA model.

In addition, in the mtDNA model, every nuclear resistance gene may aggregate by a positive selection in the predisposed matrilineal pedigrees that succeed to the same pathogenic mitochondrial genome, and may be associated with the disease [55].

Recently Marchbanks et al. [56] identified a heteroplasmic mtDNA sequence variant associated with oxidative stress in SZ. Munakata et al. [57] detected mtDNA 3243A>G mutation in the post-mortem brain of one patient with SZ. Martorell et al. [58] reported a heteroplasmic missense mtDNA variant in five of six mother-offspring schizophrenic patients pairs. Although these findings should be replicated in large-sampled studies, they may suggest another direction to search for the solution of the big conundrum that remains between the epidemiology and the molecular genetics of SZ.

## Methods

### 1. Basic assumptions

To begin, we describe our basic assumptions. These assumptions represent limitations of our study.

#### An ideal human population

Here we assume a random-mating human population with a sufficiently large effective population size at equilibrium, where negative selection pressures on the susceptibility alleles for SZ are predominant and the effect of genetic drift is negligibly small. The prevalence *p* (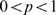) and the incidence of SZ in this ideal human population are assumed to be stable across generations through mutation-selection balance.

#### Mutation-selection balance in each risk locus

The assumption that population frequency of *each pathogenic allele* is preserved by mutation-selection balance may be too strong. Therefore, we assume here that the total of the population frequencies of the pathogenic alleles at *each risk locus* is preserved by mutation-selection balance.

#### Multifactorial threshold model

**Figure 2:**
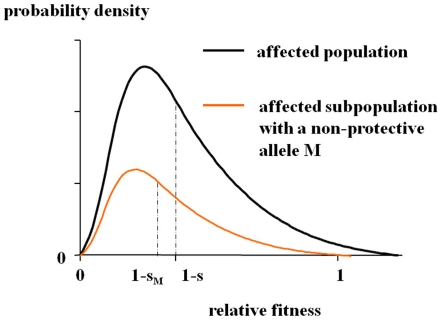
Distribution of the relative fitness in the affected population. In the multifactorial threshold model, the relative fitness as a quantitative trait in the affected population is assumed to approximately follow a gamma distribution with the mean 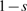. The distribution curve in the affected subpopulation with an allele *M* shifts to the right only if *M* has a strong protective effect. Thus it can be assumed that the relative fitness in the affected subpopulation with a pathogenic allele *M* approximately follows a gamma distribution with a mean not greater than 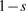 (i.e. 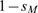; 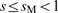).

We assume the multifactorial threshold model [1], in which quantitative traits such as liability to the disease are determined by multiple genetic and non-genetic factors including a stochastic and/or an epigenetic effect. Under this assumption, the relative fitness as a quantitative trait in the affected population is determined by multiple factors and approximately follows a gamma distribution with a mean 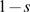 (**Figure 2**). (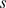 is the selection coefficient of SZ; the mean relative fitness in the normal population is defined as unity.)

The distribution curve of the fitness in the affected subpopulation with an allele *M* never shifts to the right unless *M* has a strong protective effect (i.e. an effect of elevating the *affected* carrier's fitness by reducing the severity of the disease). Since a pathogenic allele for a deleterious disease can be assumed not to elevate the affected carrier's fitness, the relative fitness in the affected subpopulation with the susceptibility allele *M* approximately follows a gamma distribution with a mean not greater than 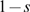 (i.e. 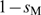; 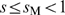).

No special assumptions else are required on the allelic structure in each locus, penetrance of each susceptibility gene, and possible interactions among the loci. It should be noted that the nuclear single major gene locus model is included as a special case in the assumptions.

### 2. Notation

#### Risk loci, two equivalent classes of alleles, and allele frequencies

Suppose that there are *n* risk loci 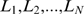 for SZ and that each locus has two equivalent classes of alleles: pathogenic and non-pathogenic. Let 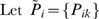 and 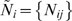 denote these classes at the risk locus 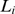. When subscripts *i* and *k* are omissible, we simply use the symbols *L*, *M*, and 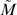 to denote a risk locus, a pathogenic allele at the risk locus, and the pathogenic class of alleles including *M* at the locus, respectively.

Let 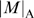, 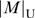, and 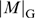 denote the frequency of an allele *M* in the affected, the unaffected and the general population, respectively. We define 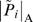, 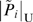, and 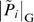 by the equations: 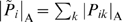, 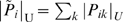, and 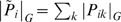.

From definition we have the following equations: for a given pathogenic allele *M*, 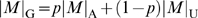, or 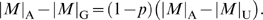
(1)


#### Cross-generational reductions of the population frequencies of the pathogenic alleles due to the decreased reproductive fitness of the affected population


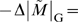
 cross-generational reduction of 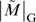 by natural selection


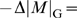
 cross-generational reduction of 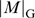 by natural selection

It may be trivial that 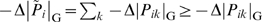.
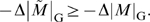
(2)


#### Mutation and mutation rates

Mutation occurs in the following directions at each risk locus 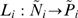, 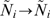, 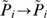, or 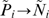. Therefore, we use the following notations:


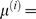
 mutation rate at the risk locus 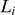, 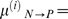 rate of mutation which occurs in the direction 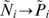 at the locus 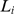, 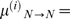 rate of mutation which occurs in the direction 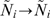 at the locus 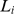, 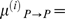 rate of mutation which occurs in the direction 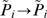 at the locus 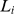, 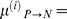 rate of mutation which occurs in the direction 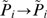 at the locus 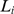, 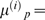 rate of mutation which produces pathogenic alleles at the locus 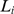, 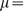 mutation rate at the risk locus *L*, 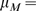 rate of mutation which produces the pathogenic allele *M* at the locus *L*, 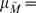 rate of mutation which produces the pathogenic alleles at the locus *L*.

From definition we have: 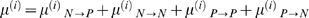, 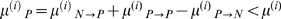, and 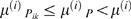.
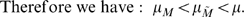
(3)Mutation−selection balance ineach risk locus implies :
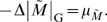
(4)


### 3. Deduction of the persistence criteria

Now we proceed to deduce the persistence criteria. From the assumptions it follows that 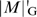, the population frequency of the pathogenic allele *M* in the next generation, is given by: 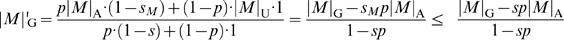. Therefore the reduction of the population frequency of the allele M per generation is: 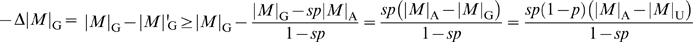
From (2), (3) and (4) it follows: 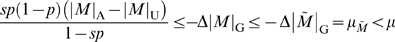.

Thus we have the first criterion for a susceptibility gene (criterion A):

#### Criterion A


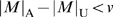
, where ν is defined by 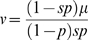.

Criterion A implies: 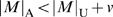. Since the odds ratio (*OR*) of the allele *M*, defined by 
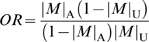
, is monotonically increasing for 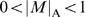, it may be trivial: if 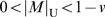, 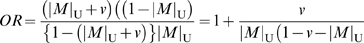.

Thus we have the second criterion for a susceptibility gene (criterion B):

#### Criterion B

If 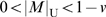, 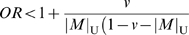.

Since criterion A also implies 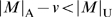 and *OR* is monotonically decreasing for 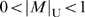, we can easily see: if 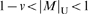,
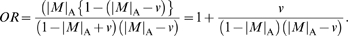


It should be noted that if 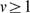 holds the persistence criteria are always fulfilled.

### 4. Numerical estimates of parameters in SZ genetics

It is now known that mutation rates on autosomes and X chromosomes almost always fall within the range of 10^−6^ to 10^−4^ per locus per generation (usually 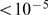; one generation  = 20 years) [59], [60]. Advancing parental ages could elevate the mutation rate [61]. Although it seems to increase as an exponential of the parental age in some loci, it can be approximated by a linear function of the parental age at least under 30 years for maternal age and under 40 years for paternal age [61]. On the other hand, large sampled cohort studies in Israel, Sweden and Denmark show that the mean age of parents in the general population is ∼28 years for mothers and ∼31 years for fathers; the mean age of both parents is <29.6 years [62], [63]. Therefore we can assume:
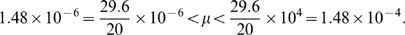



We can know the values of the parameters *p* and *s* from the epidemiological studies. Among the many epidemiological studies on the fertility of SZ, the cohort study by Haukka et al. [17] is the largest in sample size (N = 870,093) and the lowest in sampling bias. They comprised all births in Finland during 1950–1959 and followed up through the National Hospital Discharge Register for Hospitalizations between 1969 and 1992. Estimated values for *p* and *s* are 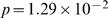 and 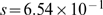. Thus, we have: 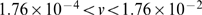.

The estimated value of 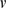 for SZ may be remarkably small. This sums up the epidemiological characteristics of SZ which discriminate it from other common diseases with genetic bases such as type 2 diabetes and most adult cancers. For those diseases 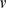 would be much greater due to much smaller *s* values because most patients with those diseases manifest after the reproductive age (>40 years). On the other hand, SZ manifests typically in adolescence or early adulthood, and specific symptoms of the disease such as an autistic way of life and bizarre behaviors reduce the reproductive fitness of the patients as has been shown by most epidemiological studies.

It should be noted that contribution of advancing parental ages to pathogenic mutations seems not very large in SZ. That is because large sampled cohort studies have shown that the proportions of older parents both in the affected and the normal populations are equally small (<7.7% and <5.5% for fathers older than 45 years in the affected and the normal populations respectively; and <9.9% and <8.7% for mothers older than 35 years) [64], [65]. In addition, the differences in the mean ages of parents between the affected and the normal individuals are not very large (<1.7 years for fathers and <0.8 year for mothers) [62], [63] even if they are statistically significant.

Some researchers have proposed the hypothesis that SZ is associated with *de novo* mutations arising in paternal germ cells [62]–[66]. It is based on the observation (‘paternal age effect’) that the risk of SZ in the offspring seems to increase as paternal age advances from 20 years to over 50 years. However, the risk of SZ was also increased in the offspring of younger men (<21 years) [62], [63], [66] as well as in the offspring of younger women (<20 years) [63]. Therefore, major roles of paternally derived mutations in SZ seem to remain unsubstantiated. Indeed, no available data can exclude the possibility that the ‘paternal age effect’ on the risk of SZ may be due to putative maternal factors; while women in many countries today may be usually supposed to bear children after the age of 20 years or to marry much older men only when the men have socio-economic benefits, predisposed women might bear children before the age of 20 years or choose too young or too old men as fathers of their children even if the men have no socio-economic benefits.

### 5. Validity-testing of the candidate genes in the literature with the criteria

We tested whether the 111 SNPs of the top 30 genes listed in the meta-analyses at SZGene (http://www.schizophreniaforum.org/res/szgene/default.asp) [30] meet the persistence criteria. Since SZGene is being periodically up-dated, we used the version on 10^th^ August, 2009. Based on the genotype distributions in meta-analyses, allele frequencies and the case-control differences were calculated. We also tested the top 100 SNPs listed in a recent GWAS by Need et al. [33] as well as the common variants reported in the latest GWASs by Shi et al. [31], by Stefasson et al. [34], and by The International Schizophrenia Consortium [32].

### 6. Power and sample size estimation in case-control association studies for SZ

Let 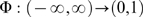 be the cumulative distribution function of the standard normal curve and let 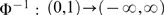 be its inverse function. The upper *β* point of the standard normal curve is given by 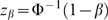 and the two sided *α* point by 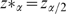. In a case-control association study of a single variant *M* with sample size 2*N* (*N* cases + *N* controls) at a significance level *α*, the power 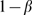 is given by 
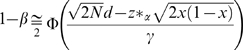
, or 
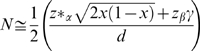
, where *x*, 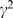, and *d* are defined by the equations: 
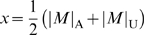
, 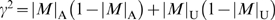, and 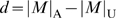. [67], [68]

Since the criterion A (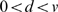) warrants 
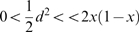
 for 0.1<*x*<0.9, we have: 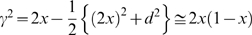, or 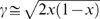. Thus we have: 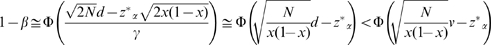, and 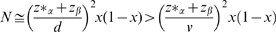 for 0.1<*x*<0.9.

We calculated the power of the association study for sample sizes *N* = 10000, 20000, 50000, 100000 under three levels of mutation rates. For the calculation of *N* required in the association study for a single allele, we assume: 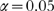
0.05 and 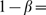
0.95, 0.8, 0.1. For the calculation of N required in GWAS, we assume: 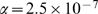 and 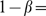
0.95, 0.8, 0.1.

## References

[1] Risch NJ (2000). Searching for genetic determinants in the new millennium.. Nature.

[2] Tandon R, Keshavan MS, Nasrallah HA (2008). Schizophrenia, “just the facts” what we know in 2008. 2. Epidemiology and etiology.. Schizophr Res.

[3] Jablensky AV, Hirsch SR, Weinberger DR (1995). Schizophrenia: the Epidemiological Horizon.. Schizophrenia.

[4] Harrison G, Cooper JE, Gancarczyk R (1991). Changes in the administrative incidence of schizophrenia.. Br J Psychiatry.

[5] Osby U, Hammer N, Brandt L, Wicks S, Thinsz Z (2001). Time trends in first admissions for schizophrenia and paranoid psychosis in Stockholm County, Sweden.. Schizophr Res.

[6] Essen-Möller E (1935). Untersuchungen über die Fruchtbarkeit gewisser Gruppen von Geisteskranken.. Acta Psychiatr Neurol.

[7] Böök JA (1953). A genetic and neuropsychiatric investigation of a North-Swedish population.. Acta Genet et Stat Med.

[8] Slater E, Hare H, Price J (1971). Marriage and fertility of psychiatric patients compared with national data.. Soc Biol.

[9] Larson CA, Nyman GE (1973). Differential fertility in schizophrenia.. Acta Psychiatr Scand.

[10] Ødegård Ø (1980). Fertility of psychiatric first admissions in Norway, 1936-1975.. Acta Psychiatr Scand.

[11] Haverkamp F, Propping P, Hilger T (1982). Is there an increase of reproductive rates in schizophrenics? I. Critical review of the literature.. Arch Psychiatr Nervenkr.

[12] Nanko S, Moridaira J (1993). Reproductive rates in schizophrenic outpatients.. Acta Psychiatr Scand.

[13] Fananas L, Bertranpetit J (1995). Reproductive rates in families of schizophrenic patients in a case-control study.. Acta Psychiatr Scand.

[14] Bassett AS, Bury A, Hodgkinson KA, Honer WG (1996). Reproductive fitness in familial schizophrenia.. Schizophr Res.

[15] Nimgaonkar VL (1998). Reduced fertility in schizophrenia: here to stay?. Acta Psychiatr Scand.

[16] McGrath JJ, Hearle J, Jenner L, Plant K, Drummond A (1999). The fertility and fecundity of patients with psychoses.. Acta Psychiatr Scand.

[17] Haukka J, Suvisaari J, Lonnqvist J (2003). Fertility of patients with schizophrenia, their siblings, and the general population: a cohort study from 1950 to 1959 in Finland.. Am J Psychiatry.

[18] Crow TJ (1995). A Darwinian approach to the origin of psychosis.. Brit J Psychiatry.

[19] Brüne M (2004). Schizophrenia – an evolutionary enigma?. Neurosci Biobehav Rev.

[20] Keller CK, Miller G (2006). Resolving the paradox of common, harmful, heritable mental disorders: Which evolutionary genetic models work best?. Behav Brain Sci.

[21] Huxley J, Mayr E, Osmond H, Hoffer A (1964). Schizophrenia as a genetic morphism.. Nature.

[22] Karlsson JL (1974). Inheritance of schizophrenia.. Acta Psychiatr Scand.

[23] Kinney DK, Richards R, Lowing PA, LeBranc D, Zimbalist ME (2001). Creativity in offspring of schizophrenic and control parents: an adoption study.. Creativity Research Journal.

[24] Shaner A, Miller G, Mintz J (2004). Schizophrenia as one extreme of a sexually selected fitness indicator.. Schizophr Res.

[25] Lindelius R (1970). A study of schizophrenia. A clinical, prognostic and family investigation.. Acta Psychiatr Scand.

[26] Buck C, Hobbs GE, Simpson H, Winokur JM (1975). Fertility of the sibs of schizophrenic patients.. Brit J Psychiatry.

[27] Rimmer J, Jacobsen B (1976). Differential fertility of adopted schizophrenics and their half-siblings.. Acta Psychiatr Scand.

[28] Erlenmeyer-Kimling L, Cancro R (1978). Fertility of psychotics: demography.. Annual Review of the Schizophrenic Syndrome.

[29] Svensson AC, Lichtenstein P, Sandin S, Hultman CM (2007). Fertility of first-degree relatives of patients with schizophrenia: A three generation perspective.. Schizophr res.

[30] Allen NC, Bagades S, McQueen MB, Ioannidis JPA, Kavvoura FK (2008). Systematic Meta-Analyses and Field Synopsis of Genetic Association Studies in Schizophrenia: The SZGene Database.. Nat Genet.

[31] Shi J, Levinson DF, Duan J, Sanders AR, Zheng Y (2009). Common variants on 6p.22.1 are associated with schizophrenia.. Nature (Published online 1 July 2009.

[32] The International Schizophrenia Consortium (2009). Common polygenic variation contributes to risk of schizophrenia and bipolar disorder.. Nature (Published online 1 July 2009.

[33] Need AC, Ge D, Weale ME, Maia J, Feng S (2009). A Genome-Wide Investigation of SNPs and CNVs in Schizophrenia.. PLoS Genet.

[34] Stefasson H, Ophoff RA, Steinberg S, Andreassen OA, Cichon S (2009). Common variants conferring risk of schizophrenia.. Nature (Published online 1 July 2009.

[35] Lander ES (1996). The new genomics: global view of biology.. Science.

[36] Reich DE, Lander ES (2001). On the allelic spectrum of human disease.. Trends Genet.

[37] Pritchard JK (2001). Are rare variants responsible for susceptibility to complex diseases?. Am J Hum Genet.

[38] Pritchard JK, Cox NJ (2002). The allelic architecture of human disease genes: common disease-common variant … or not?. Hum Mol Genet.

[39] Mah S, Nelson MR, DeLisi LE, Reneland RH, Markward N (2006). identification of the semaphoring receptor *PLXMA2* as a candidate for susceptibility to schizophrenia.. Mol Psychiatry.

[40] Lenz T, Morgan TV, Athanasiou M, Dain B, Reed CR (2007). Converging evidence for a pseudoautosomal cytokine receptor gene locus in schizophrenia.. Mol Psychiatry.

[41] Kirov G, Zaharieva I, Georgieva L, Moskvina V, Nikolov I (2008). A genome-wide association study in 574 schizophrenia trios using DNA pooling.. Mol Psychiatry.

[42] Shifman S, Johannesson M, Bronstein M, Chen SX, Collier DA (2008). Genome-wide association identifies a common variant in the reelin gene that increases the risk of schizophrenia only in women.. PLoS Genet.

[43] O'Donovan MC, Craddock N, Norton N, Williams H, Peirce T (2008). Identification of loci associated with schizophrenia by genome-wide association and follow up.. Nat Genet.

[44] Sullivan PF, Lin D, Tzeng JY, van den Oord E, Perkin D (2008). Genomewide association study for schizophrenia in the CATIE study: results of stage I.. Mol Psychiatry.

[45] Sanders AR, Duan J, Levinson DF, Shi J, He D (2008). No significant association of 14 candidate genes with schizophrenia in a large European ancestry sample: implication for psychiatric genetics.. Am J Psychiatry.

[46] Friedman JI, Vrijenhoek T, Markx S, Janssen IM, van der Vliet WA (2008). *CNTNAP2* gene dosage variation is associated with schizophrenia and epilepsy.. Mol Psychiatry.

[47] Kirov G, Gumus D, Chen W, Norton N, Georgieva L (2008). Comparative genome hybridization suggests a role for *NRXN1* and *APBA2* in schizophrenia.. Hum Mol Genet.

[48] Stefasson H, Rujescu D, Cichon S, Pietiläinen OP, Ingason A (2008). Large recurrent microdeletions associated with schizophrenia.. Nature.

[49] The International Schizophrenia Consortium (2008). Rare chromosomal deletions and duplications increase risk of schizophrenia.. Nature.

[50] Walsh T, McClean JM, McCarthy SE, Addington AM, Pierce SB (2008). Rare structural variants disrupt multiple genes in neurodevelopmental pathways in schizophrenia.. Science.

[51] Xu B, Roos JL, Levy S, van Rensburg EJ, Gogos JA (2008). Strong association of de novo copy number mutations with sporadic schizophrenia.. Nat Genet.

[52] Repping S, van Daalen SKM, Brown LG, Korver CM, Lange J (2006). High mutation rates have driven extensive structural polymorphism among human Y chromosomes.. Nat Genet.

[53] Sigurđardóttir S, Helgason A, Gulcher JR, Stefansson K, Donnely P (2000). The mutation rate in the human mtDNA control region.. Am J Hum Genet.

[54] Doi N, Hoshi Y (2007). Persistence problem in schizophrenia and mitochondrial DNA.. Am J Med Genet Part B.

[55] Doi N, Itokawa M, Hoshi Y, Arai M, Furukawa A (2007). A resistance gene in disguise for schizophrenia?. Am J Med Genet Part B.

[56] Marchbanks RM, Ryan M, Day IN, Owen M, McGuffin P (2003). A mitochondrial DNA sequence variant associated with schizophrenia and oxidative stress.. Schizophr Res.

[57] Munakata K, Iwamoto K, Bundo M, Kato T (2005). Mitochondrial DNA 3243A>G mutation and increased expression of *LARS2* gene in the brains of patients with bipolar disorder and schizophrenia.. Biol Psychiatry.

[58] Martorell L, Segués T, Folch G, Valero J, Joven J (2006). New variants in the mitochondrial genomes of schizophrenic patients.. Eur J Hum Genet.

[59] Vogel F, Motulsky AG (1997). Human genetics: problems and approaches..

[60] Nachman MW, Crowell SL (2000). Estimate of the mutation rate per nucleotide in humans.. Genetics.

[61] Risch N, Reich EW, Wishnick MM, McCarthy JG (1987). Spontaneous mutation and parental age in humans.. Am J Hum Genet.

[62] Malaspina D, Harlap S, Fennig S, Heiman D, Nahon D (2001). Advancing paternal age and the risk of schizophrenia.. Arch Gen Psychiatry.

[63] El-Saadi O, Pedersen CB, McNeil TF, Saha S, Welham J (2004). Paternal and maternal age as risk factors for psychosis: findings from Denmark, Sweden and Australia.. Schizophr Res.

[64] Zammit S, Allebeck P, Dalman C, Lundberg I, Hemmingson T (2003). Paternal age and risk for schizophrenia.. Brit J Psychiatry.

[65] Byrne M, Agerbo E, Ewald H, Eaton WW, Mortensen PB (2003). Parental age risk of schizophrenia. A case-control study.. Arch Gen Psychiatry.

[66] Sipos A, Rasmussen F, Harrison G, Tynelius P, Lewis G (2004). Paternal age and schizophrenia; a population based cohort study.. BMJ.

[67] Ohashi J, Yamamoto S, Tsuchiya N, Hatta Y, Komata T (2001). Comparison of statistical power between 2×2 allele frequency and allele positivity tables in case-control studies of complex disease genes.. Ann Hum Genet.

[68] Ohashi J, Tokunaga K (2002). the expected power of genome-wide linkage disequilibrium testing using single nucleotide polymorphism markers for detecting a low-frequency disease variant.. Ann Hum Genet.

